# Towards a national strategy for digital pathology in Switzerland

**DOI:** 10.1007/s00428-022-03345-0

**Published:** 2022-05-27

**Authors:** Andrew Janowczyk, Daniel Baumhoer, Stefan Dirnhofer, Rainer Grobholz, Anja Kipar, Laurence de Leval, Doron Merkler, Olivier Michielin, Holger Moch, Aurel Perren, Sven Rottenberg, Laura Rubbia-Brandt, Mark A. Rubin, Christine Sempoux, Markus Tolnay, Inti Zlobec, Viktor Hendrik Koelzer, the Swiss Digital Pathology Consortium (SDiPath)

**Affiliations:** 1 Department of Oncology, Lausanne University Hospital, Lausanne, Switzerland; 2 Biomedical Engineering Department, Case Western Reserve University, Cleveland, OH USA; 3 Institute of Medical Genetics and Pathology, University Hospital Basel, University of Basel, Basel, Switzerland; 4 Medical Faculty, University of Zurich, Zurich, Switzerland; 5 Institute of Pathology, Kantonsspital Aarau, Aarau, Switzerland; 6 Institute of Veterinary Pathology, Vetsuisse Faculty, University of Zurich, Zurich, Switzerland; 7 Institute of Pathology, Department of Laboratory Medicine and Pathology, Lausanne University Hospital and Lausanne University, Lausanne, Switzerland; 8 Department of Pathology and Immunology, University of Geneva, Geneva, Switzerland; 9 Division of Clinical Pathology, Diagnostic Departement Geneva University Hospital and Faculty of Medicine of Geneva, Geneva, Switzerland; 10 Precision Oncology Center, Department of Oncology, Lausanne University Hospital, Lausanne, Switzerland; 11 Molecular Modelling Group, Swiss Institute of Bioinformatics (SIB), Lausanne, Switzerland; 12 Department of Pathology and Molecular Pathology, University of Zurich and University Hospital Zurich, Raemistrasse 100, CH-8091 Zurich, Switzerland; 13 Comprehensive Cancer Center Zurich, Zurich, Switzerland; 14 Institute of Pathology, University of Bern, Murtenstrasse 31, CH-3008 Bern, Switzerland; 15 Institute of Animal Pathology, Vetsuisse Faculty, University of Bern, Bern, Switzerland; 16 Bern Center for Precision Medicine, University of Bern, Bern, Switzerland; 17 Department for BioMedical Research, Precision Oncology Laboratory, University of Bern, Bern, Switzerland

**Keywords:** Precision medicine, Biomedical research, Pathology, Image analysis, Artificial intelligence

## Abstract

Precision medicine is entering a new era of digital diagnostics; the availability of integrated digital pathology (DP) and structured clinical datasets has the potential to become a key catalyst for biomedical research, education and business development. In Europe, national programs for sharing of this data will be crucial for the development, testing, and validation of machine learning–enabled tools supporting clinical decision-making. Here, the Swiss Digital Pathology Consortium (SDiPath) discusses the creation of a Swiss Digital Pathology Infrastructure (SDPI), which aims to develop a unified national DP network bringing together the Swiss Personalized Health Network (SPHN) with Swiss university hospitals and subsequent inclusion of cantonal and private institutions. This effort builds on existing developments for the national implementation of structured pathology reporting. Opening this national infrastructure and data to international researchers in a sequential rollout phase can enable the large-scale integration of health data and pooling of resources for research purposes and clinical trials. Therefore, the concept of a SDPI directly synergizes with the priorities of the European Commission communication on the digital transformation of healthcare on an international level, and with the aims of the Swiss State Secretariat for Economic Affairs (SECO) for advancing research and innovation in the digitalization domain. SDPI directly addresses the needs of existing national and international research programs in neoplastic and non-neoplastic diseases by providing unprecedented access to well-curated clinicopathological datasets for the development and implementation of novel integrative methods for analysis of clinical outcomes and treatment response. In conclusion, a SDPI would facilitate and strengthen inter-institutional collaboration in technology, clinical development, business and research at a national and international scale, promoting improved patient care via precision medicine.

## Background

Globally, despite significant advancements in medical technology, reaching a definitive diagnosis for many diseases still requires the microscopic evaluation of clinical tissue samples by surgical pathologists. In 2019, the European Union (EU) approved the usage of whole slide scans for such primary diagnoses, wherein routine glass histopathology slides are digitized and presented to pathologists for review on computer monitors. The potential of this digital pathology (DP) approach represents a major inflection point for both research and clinical workflows, since once slides are in a digital format, they become amenable to digital transfer and computational analysis.

Research and development by industry and academic institutions have shown that these DP images can be analysed by sophisticated machine learning algorithms to enable (a) more precise characterization of the histological spectrum of disease, and (b) data mining of specific features for clinically relevant biomarkers [1, 2]. By supporting diagnostic, prognostic and therapy response predictions, DP research is uniquely staged to significantly contribute towards the goal of precision medicine, wherein treatment plans are determined on a per-patient basis as a result of analysing continually growing retrospective cohorts. These image-based approaches benefit from being orders of magnitude faster and more cost-effective than their genetic assay-based counterparts, potentially enabling a more complete characterization of tumour heterogeneity [3]. Additionally, DP methods are tissue non-destructive, facilitating longitudinal studies of algorithm improvement as the understanding of disease pathophysiology and progression grows. Furthermore, defined and fully investigated case material will enable the validation and comparative assessment of animal models of specific entities/diseases, fostering their meaningful and effective use in potentially novel treatment approaches and thereby improving their translatability.

**Fig. 1:**
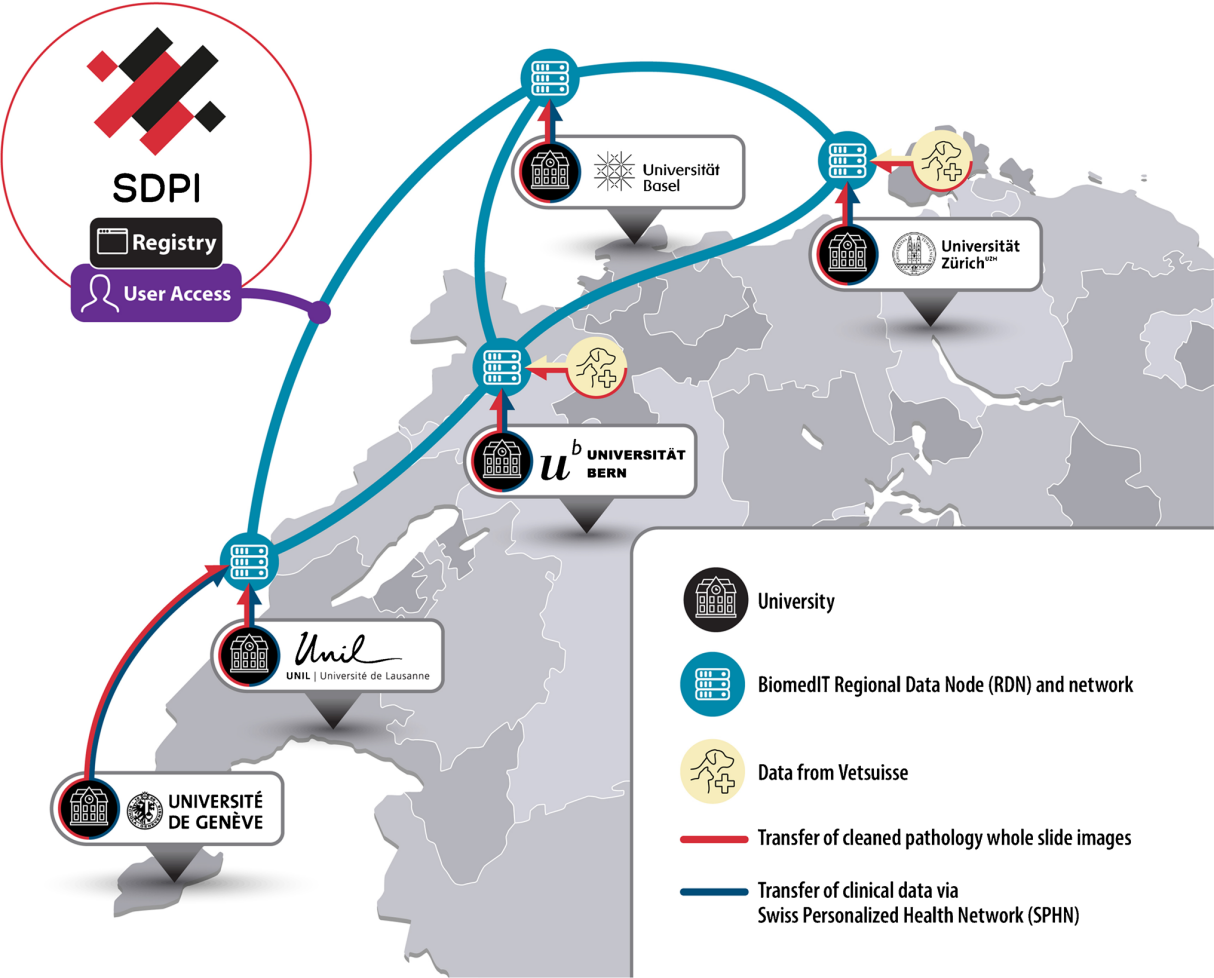
Network map for SDPI. SDPI will establish a unified national DP network bringing together the Swiss Personalized Health Network (SPHN) with Swiss university hospitals. SDPI envisages that clinical data, veterinary pathology data (Vetsuisse) and whole slide pathology images generated at the university hospitals will be provided to the SPHN for storage and countrywide access on the BioMedIT Network with subsequent inclusion of cantonal and private institutions. Users will be able to search the SDPI network through a centralized registry for the formation of virtual research cohorts and clinical trials

In Switzerland, and inclusive of a broader view in the European Union, there is an emerging opportunity to develop a unified national DP network consisting of both public and private institutions [4, 5]. Each site could contribute digital slides in synergy with existing roadmaps for digitalization (Fig. 1). These digital slides could then be made available, via appropriate privacy-preserving safeguards, to other clinicians and researchers within Switzerland. A Swiss Digital Pathology Infrastructure (SDPI) would thus organically facilitate (a) multi-site clinical trials and (b) implementation of tools for increased diagnostic accuracy, quality and patient safety, while simultaneously unlocking the hidden value of DP images by (c) advancing the utilization of artificial intelligence (AI) towards improved patient-level diagnosis, prognosis and therapy response prediction [2, 3, 6, 7].

This call for a national infrastructure is in concert with other recent national investments by the UK [8], Germany [9], Sweden [10] and the Netherlands [11] as well as the European Union Innovative Medicines Initiative (IMI) [12] (Table 1). These investments have already proven fruitful in terms of improved patient care, intellectual property generation, education, employment, start-up generation and granting of competitive EU funding. SDiPath, an organization consisting of over 130 Swiss-based members whose primary mandate is to facilitate the implementation and development of DP in Switzerland, is hoping to inspire a similar investment within Switzerland.

**Table 1:** Key digital pathology infrastructures in Europe

DP network	Country/area	URL/reference
EcosysteM for Pathology Diagnostics with AI Assistance (EMPAIA)	Germany	https://www.empaia.org/
Pathology Image Data Lake for Analytics Knowledge & Education (PathLAKE)	UK	https://www.pathlake.org/
Pathology Image Exchange (PIE)	The Netherlands	https://www.palga.nl/professionals/pie.html
Swedish Digital Pathology Program	Sweden	Asa SL, Boden AC, Treanor D, Jarkman S, Lundstrom C, Pantanowitz L (2019) 2020 Vision of Digital Pathology in Action J Pathol Inform 10:27. https://doi.org/10.4103/jpi.jpi_31_19
BIGPICTURE—a central repository of digital pathology slides to boost the development of artificial intelligence	European Union, Innovative Medicines Initiative (IMI)	https://bigpicture.eu/

## A national roadmap for digitalization

A SDPI would promote digital diagnostic workflows on a national level by establishing five components: (a) data creation, (b) data storage, (c) data sharing, (d) data enhancement, and (e) data computing, all developed in line with the data principles of *F*indability, *A*ccessibility, *I*nteroperability, *R*eusability (FAIR).

### Data creation

SDPI aims for a highly standardized environment for data creation, storage, and retrieval. This will enable improved cross-site data sharing and algorithm development, with linkage to national and international data sharing programs in a cost-efficient manner.

### Data storage

Images will be stored on-site, in line with established hospital privacy, redundancy, and backup requirements. National guidelines which are currently being developed by a dedicated SDiPath Working Group are expected by mid 2022. Storage infrastructure would be designed in a scalable manner, such that as data is routinely created and additional storage is required, supplemental capacity can seamlessly be brought online.

### Data sharing

Approved users should be able to remotely query the SDPI in a privacy-preserving manner, enabling the estimation of cohort sizes for research projects and clinical trials. This information will be critical to obtain ethics approval for the release of coded data in accordance with established biobanking protocols and national regulations. Connection to the growing Swiss Personalized Health Network (SPHN) is envisaged to achieve multi-modal integration of health data for research purposes and integration in clinical trials [13]. Synoptic reporting, previously developed under the multi-institutional PathoLink project, would facilitate the standardization of datasets and cross-language translation of health reports [6].

### Data enhancement

Annotations are needed for developing AI algorithms, biomarkers and tools. These annotations are often extremely time-consuming to collect, as only topic experts are able to create and validate their accuracy. The DP community has witnessed a significant reduction in experimental execution times via sharing of these annotations and associated tools [14, 15]. Building on this, a SDPI should store these high-value annotations with appropriate metadata, transforming a DP slide repository from solely a collection of images into a genuinely useful, queryable, reusable, information-rich resource abiding by FAIR principles.

### Data computing

**Fig. 2:**
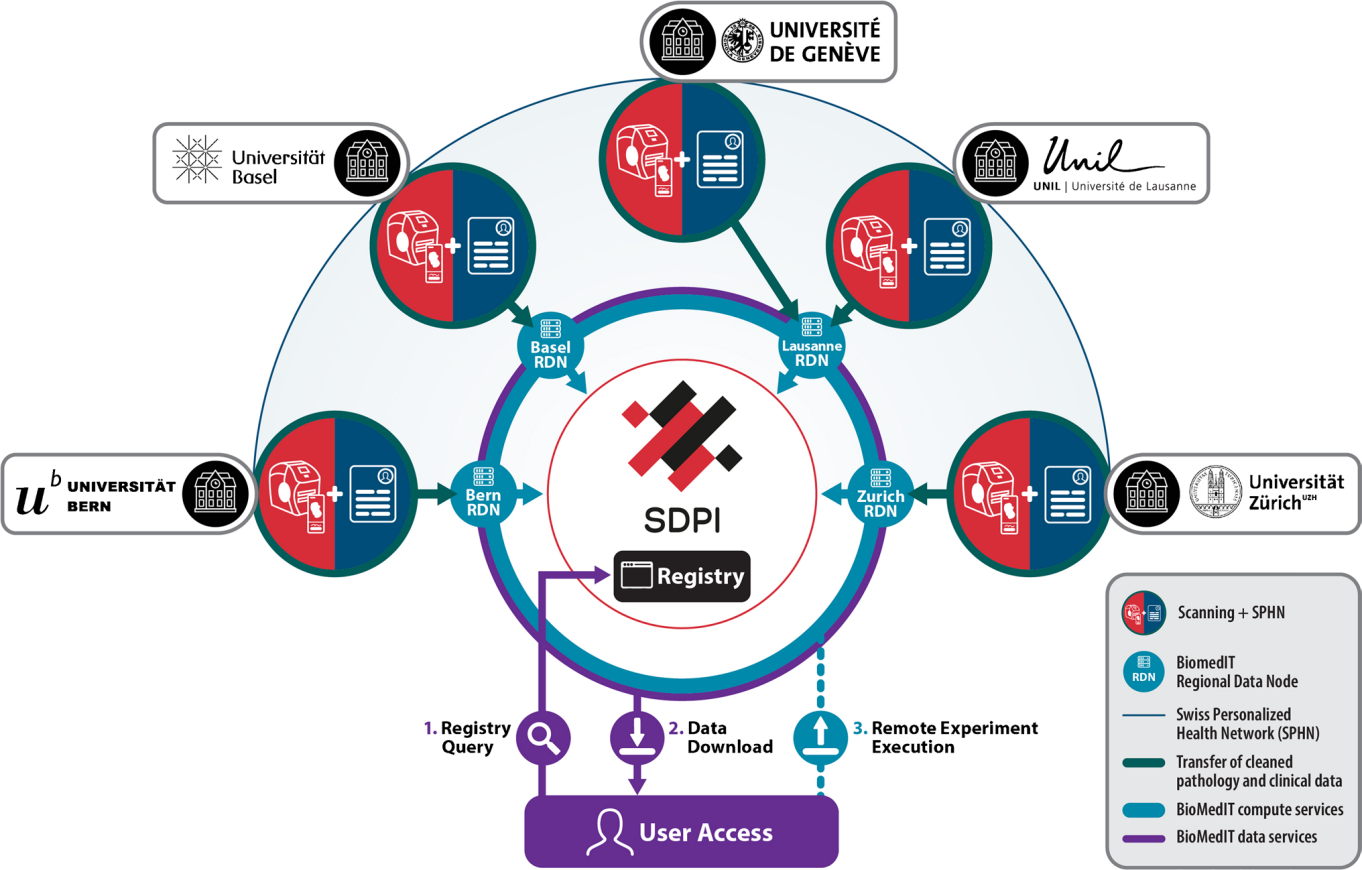
Illustration of expected SDPI data collection, processing, and user experience. SDPI data is generated at each partner institute through (a) digitalization of routine histology sections in SDPI scanner infrastructure to generate SDPI WSIs and (b) collection of the associated coded and standardized pathology data for each case. SDPI data is provided to the Regional Data Service Centre of the university hospitals, is unified with existing SPHN datasets and is subsequently pushed to the nearest Regional Data Node (RDN) of the BioMedIT network. Relevant clinical data and cohort characteristics are aggregated in the SDPI registry extension, affording (1) the opportunity for a single-access point for dataset querying by the user, after which (2) the user can view, process and download the SDPI data in a distributed manner from the respective RDN nodes where the data resides. Alternatively, (3) the user can upload their containerized experiment to the BioMedIT computational nodes where it is executed remotely

SDPI will provide high quality–coded, semantically structured and harmonized DP datasets for research purposes organized in the distributed BioMedIT data repository and made findable, accessible, interoperable and retrievable through a centralized registry with access control [16] (Fig. 2). Through the integration of SDPI with the BioMedIT infrastructure, researchers will be able to access the compute capabilities of the BioMedIT high-performance computing nodes for off-site processing of SDPI datasets through a unified access point. The existing BioMedIT nodes contain specialized computational infrastructure, such as the graphical processing units (GPUs) often employed in DP research experiments, and thus allow a fee-for-service model for remote analysis of cohorts by researchers who may not have access to such resources locally. Similar to popular cloud service providers such as Amazon Web Services (AWS), users will be able to containerize their code and software environments for transfer and execution on the node, greatly easing the development and deployment burden by allowing for local buildout and testing before remote execution. Additionally, in line with current technical developments, the data sharing infrastructure will be designed to support sophisticated next-generation experimental setups, such as federated machine learning. These approaches often train models across multiple decentralized servers, without the need for data exchange or centralization, thus greatly minimizing local hardware and storage needs and potentially facilitating the preservation of data privacy.

### Future growth

Determination of minimal hardware, software, technical, and ethical requirements is crucial to enable smaller public and private hospitals with limited resources to contribute to the development of a SDPI. From the onset, a “plug and play” design must be considered, enabling new data producers to rapidly connect to the infrastructure in a standardized way, limiting costly re-development and one-off solutions.

## National and international strategic context

Switzerland has large vested interests in “Personalized Medicine” (e.g. National Support Initiatives), and DP represents a highly synergistic and complementary approach to (a) genomic sequencing cores at university hospitals, (b) national networks to support data science established by the SPHN, (c) the Swiss Institute of Bioinformatics, and (d) the Swiss Biobanking Platform. On a European level, the ability to share resources (data, expertise, computing and storage capacities) is seen as a key driver of greater research insights, impactful economic development opportunities and better patient care [17].

DP affords the opportunity for significantly improved efficiency in terms of clinical care and disease research and in the technical development of diagnostic medical tools. These facets each require large quantities of data for both development and validation and yet no infrastructure in Switzerland exists to provide it. A SDPI would overcome this hurdle yielding benefits such as (a) decreased waiting times for pathological evaluation and improved (b) quality, (c) safety, (d) speed, and (e) specificity in medical assessments. Furthermore, a deployment infrastructure will enable sharing of clinically validated biomarkers, tools, and algorithms. This will thus ensure continual high-quality medical care for Swiss patients, while countering the anticipated shortage of pathologists during the next decade by increasing their efficiency.

In that light, a number of university and public hospitals have already begun the digital transformation, in total having scanned well over a million slides, at an increasing rate of over 50k slides per year [4, 5]. SDPI will further extend this process, while imparting a consolidated interface for interaction with this data by researchers. Furthermore, these new capabilities will harmonize with SPHN efforts, such that a fully unified pan-Switzerland medical infrastructure is formed.

## National and international importance

The gold standard of clinical trial design requires large retrospective and prospective cohorts. Similar to other countries in Europe with limited populations, it is challenging for any isolated centre in Switzerland to recruit enough patients to conduct such trials. This places Switzerland at a disadvantage as compared to higher population countries, whose high-volume hospitals have been able to position themselves as lead innovators due to their access to large numbers of patients. A SDPI resolves this issue by allowing for the seamless merging of all patients across Switzerland into a virtual “single cohort”, enabling a competitive advantage that surpasses even those of large-single institute clinics. An attractive collaborative opportunity is thus created between Swiss Hospitals and Clinical Research Organisations which are both eager to conduct clinical trials, while at the same time are also limited in access to suitable patient populations. A more direct, cost-efficient collaboration between these two types of research entities, not only enables patients to be better matched to potentially life-saving treatment, but also facilitates the discovery of biomarkers to heavily reduce ineffective overtreatment and thus limit long-term potential toxic side effects of many therapies.

## Conclusion

Taken together, the high quality of care available in Switzerland, paired with its world-class precision medicine, cancer research and basic and translational science teams, creates an especially timely opportunity to genuinely improve patient care in the near term by the development of a highly synergistic nationwide Swiss Digital Pathology Infrastructure.

## Abbreviations

*DP*: Digital pathology
*WSI*: Whole slide image
*LIS*: Laboratory information system
*SDiPath*: Swiss Digital Pathology Consortium
